# Supplementary material for: Sleep Quality Disturbances Are Associated with White Matter Alterations in Veterans with Post-Traumatic Stress Disorder and Mild Traumatic Brain Injury
Source: J Clin Med. 2023 Mar 6;12(5):2079. doi: 10.3390/jcm12052079 (PMC10004675; doi:10.3390/jcm12052079)
Supplement: Supplementary file 1 [file jcm-12-02079-s001.zip › jcm-2259161-supplementary.pdf]

Journal of
Clinical Medicine

MDPI

Supplementary Tables

Supplementary Table S1. Sample characteristics: Group comparisons

		**PTSD vs. mTBI vs. Comorbid PTSD+mTBI vs. No history of PTSD or mTBI**	Post-hoc **PTSD vs. mTBI**	Post-hoc **PTSD vs. Comorbid PTSD+mTBI**	Post-hoc **mTBI vs. Comorbid PTSD+mTBI**	Post-hoc **PTSD vs. No history of PTSD or mTBI**	Post-hoc **mTBI vs. No history of PTSD or mTBI**	Post-hoc **Comorbid PTSD+mTBI vs. No history of PTSD or mTBI**
		**ANCOVA**						
		F*(df), *p	***p***	***p***	***p***	***p***	***p***	***p***
**Age (years)**		.25(3, 176), .859	.473	.979	.425	.735	.741	.717
**Education (years)**		2.07(3, 175), .106	.058	.171	.311	**.029**	.761	.175
**Number of OEF/OIF deployments**		.09(3,175), .968	.920	.883	.809	.749	.839	.627
**Number of other stressful deployments**		1.59(3, 175), .195	.387	.404	.090	.390	.990	.097
**Total duration of OEF/OIF deployments (months)**		.03(3, 175), .995	.820	.819	.948	.946	.888	.911
**Total duration of other deployments (months)**		1.30(3, 175), .275	.205	.742	.084	.397	.720	.218
**Number of lifetime mTBIs**		16.51(3, 175), **<.001**	-	-	.123	-	-	-
**Lifetime mTBI burden**		17.47(3,175), **<.001**	-	-	.075	-	-	-
**Total CAPS**		47.40(3, 175), **<.001**	**<.001**	**.001**	**<.001**	**<.001**	.555	**<.001**
		**Chi-square or Fisher's exact test**						
		**χ^2(df), *p***	***p***	***p***	***p***	***p***	***p***	***p***
**Ethnicity**	American Indian or Alaska Native	2.04(3), 1.000	-	1.000	1.000	-	-	1.000
	Asian	1.69(3), .729	1.000	.494	1.000	1.000	-	1.000
	Black	3.34(3), .318	1.000	.373	.395	.287	.235	.596
	Hispanic or Latino	1.36(3), .713	.741	.772	.530	.698	.419	.732
	Native Hawaiian or Pacific Islander	-	-	-	-	-	-	-
	White	5.71(3), .137	.972	.211	.261	**.039**	.075	.237
	Unknown	3.85(3), .478	1.000	.288	-	1.000	-	-
**Service branch**	Army	4.33(3), .226	.176	.117	.860	1.000	.249	.240
	Army National Guard	4.51(3), .212	.967	.184	.233	.523	.585	.062
	Air Force	1.15(3), .844	.557	.673	.637	1.000	1.000	1.000
	Air Force National Guard	.99(3), .938	1.000	.625	1.000	1.000	1.000	1.000
	Coast Guard	-	-	-	-	-	-	-
	Navy	1.06(3), .838	1.000	.673	1.000	1.000	1.000	.581
	Marines	6.69(3), .080	1.000	.059	.198	.700	.668	**.046**
	Reserves	6.74(3), .070	.103	**.013**	1.000	.422	.407	.282
	National Guard, branch unknown	5.44(3), .080	1.000	1.000	1.000	.146	.102	.051
**Psychiatric diagnoses**	Lifetime substance use disorder	12.50(3), **.005**	.503	.063	**.016**	.195	.555	**.002**
	Lifetime mood disorder	12.77(3), **.005**	.073	.409	**.009**	**.045**	1.000	**.005**
	Lifetime anxiety disorder	1.81(3), 6.38	.334	.297	.761	.508	1.000	1.000
**mTBI**	Military mTBI	60.09(3), **<.001**	-	-	**.004**	-	-	-

Note. SD, Standard deviation; PTSD, Post-traumatic stress disorder; mTBI, Mild traumatic brain injury; OEF, Operation Enduring Freedom; OIF, Operation Iraqi Freedom; CAPS, Clinician Administered PTSD Scale; PSQI, Pittsburgh Sleep Quality Index; PSQI 3-factor structure, The PSQI subscales sleep efficiency, perceived sleep quality & daily disturbances refer to the Pittsburgh Sleep Quality Index 3-Factor structure. All ANCOVA's were corrected for age.

Supplementary Table S2. Association between PTSD symptom severity and sleep quality

			**PTSD**				**mTBI**				**Comorbid PTSD+mTBI**				**No history of PTSD or mTBI**			
			**Regression**				**Regression**				**Regression**				**Regression**			
**DV**		**IV**	***β***	***t***	***f²***	***p****	***β***	***t***	***f²***	***p****	***β***	***t***	***f²***	***p****	***β***	***t***	***f²***	***p****
**PSQI Global**		Total CAPS	.49	3.40	.32	**.002**	.62	3.68	.61	**.001**	.53	5.82	.38	**<.001**	-.15	-.64	.02	.531
**PSQI 3-factor structure**	Sleep efficiency	Total CAPS	.32	2.07	.11	.046	.51	2.79	.36	**.011**	.22	2.17	.05	.033				
	Perceived sleep quality	Total CAPS	.45	2.99	.25	**.005**	.40	2.03	.18	.055	.51	5.55	.34	**<.001**				
	Daily disturbances	Total CAPS	-48	3.25	.30	**.003**	.47	2.57	.29	.018	.55	6.16	.42	**<.001**				

Note. PTSD, Post-traumatic stress disorder; mTBI, Mild traumatic brain injury; DV, Dependent variable; IV, Independent variable; *β*, Standardized beta coefficient; f^2, effect size; CAPS, Clinician Administered PTSD Scale; PSQI, Pittsburgh Sleep Quality Index; The PSQI subscales sleep efficiency, perceived sleep quality & daily disturbances refer to the Pittsburgh Sleep Quality Index 3-Factor Structure. p^* corrected for multiple comparisons as outlined in Figure 1.

Supplementary Table S3. Association between sleep quality and white matter FA_T

		**PTSD**		**mTBI**		**Comorbid PTSD+mTBI**		**No history of PTSD or mTBI**	
**DV**	**IV**	**Regression**		**Regression**		**Regression**		**Regression**	
**Right FA_T**	**PSQI Global**	***t***	***p***	***t***	***p***	***t***	***p***	***t***	***p***
**AF**		.09	.926	-.26	.797	-.25	.803	-.43	.669
**CB**		.845	.404	-.70	.491	-3.56	**<.001**	-1.35	1.92
**ILF**		.984	.332	-2.51	**.020**	-2.04	**.044**	-.62	.542
**IOFF**		.543	.590	.65	.525	-2.87	**.005**	-.75	.465
**MdLF**		-.47	.643	-1.05	.307	-2.14	**.035**	-1.88	.074
**SLF-I**		1.33	.192	-1.23	.230	-2.49	**.015**	-.25	.806
**SLF-II**		.284	.805	-.72	.481	-1.31	.195	-1.25	.277
**SLF-III**		.034	.973	-1.39	.178	-1.01	.318	-.15	.884
**UF**		.126	.900	-.83	.418	-1.33	.188	-.43	.674
**Left FA_T**	**PSQI Global**								
**AF**		1.00	.323	-.04	.968	-1.03	.307	-1.77	.092
**CB**		-.25	.806	.59	.561	-1.963	.053	-1.46	.160
**ILF**		.678	.502	.77	.446	-2.72	**.008**	-2.56	**.019**
**IOFF**		.816	.420	-.94	.355	-3.53	**<.001**	-1.16	.260
**MdLF**		.167	.868	-1.44	.164	-3.08	**.003**	-1.79	.089
**SLF-I**		-.21	.832	-.47	.644	-2.42	**.017**	-1.49	.151
**SLF-II**		.21	.835	1.03	.315	-2.35	**.021**	-.812	.426
**SLF-III**		.61	.545	-.02	.984	-2.55	**.012**	-1.00	.336
**UF**		.43	.674	-.838	.411	-2.15	**.035**	-.138	.892
**Commissural FA_T**	**PSQI Global**								
**CC-I**		-1.12	.272	-.25	.028	-3.61	**<.001**	-.97	.343
**CC-II**		-.67	.506	-190	.071	-2.41	**.018**	-1.03	.314
**CC-III**		-.44	.661	-.97	.344	-2.14	**.035**	-1.77	.092
**CC-IV**		1.52	.139	-.86	.399	-2.52	**.013**	-.42	.680
**CC-V**		1.38	.176	-.16	.871	-2.09	**.040**	-.64	.531
**CC-VI**		.10	.918	-.225	8.24	-1.33	.189	-.48	.635
**CC-VII**		-.07	.944	-.75	.461	.23	.819	-.62	.543

Note. PTSD, Post-traumatic stress disorder; mTBI, Mild traumatic brain injury; DV, Dependent variable; IV, Independent variable; FA_T, Fractional anisotropy $_{Tissue}$; PSQI, Pittsburgh Sleep Quality Index; AF, Arcuate fasciculus, CB, Cingulum bundle; ILF; Inferior longitudinal fasciculus; IOFF, Inferior occipito-frontal fasciculus; MdLF, Middle longitudinal fasciculus; SLF, Superior longitudinal fasciculus; UF, Uncinate fasciculus; CC, Corpus callosum.

Supplementary Table S4. Association between sleep quality and whole-brain FA_T

		**PTSD**				**mTBI**				**Comorbid PTSD+mTBI**				**No history of PTSD or mTBI**			
		**Regression**				**Regression**				**Regression**				**Regression**			
**DV**	**IV**	***β***	***t***	***f^2***	***p****	***β***	***t***	***f^2***	***p****	***β***	***t***	***f^2***	***p****	***β***	***t***	***f^2***	***p****
**Whole-Brain FA_T**	PSQI Global	.06	.35	.00	.727	-.15	-.80	.02	.434	-.39	-4.04	.18	**<.001**	-.25	-1.21	.07	.241
	PSQI Sleep efficiency									.10	1.02	.01	.309				
	PSQI Perceived sleep quality									-.43	-3.86	.21	**<.001**				
	PSQI Daily disturbances									-.15	-1.46	.09	.149				

Note. PTSD, Post-traumatic stress disorder; mTBI, Mild traumatic brain injury; DV, Dependent variable; IV, Independent variable; *β*, Standardized beta coefficient; f^2, effect size; FA_T, Fractional anisotropy $_{Tissue}$; PSQI, Pittsburgh Sleep Quality Index; The PSQI subscales sleep efficiency, perceived sleep quality & daily disturbances refer to the Pittsburgh Sleep Quality Index 3-Factor Structure. p^* corrected for multiple comparisons as outlined in Figure 1.

Supplementary Figures

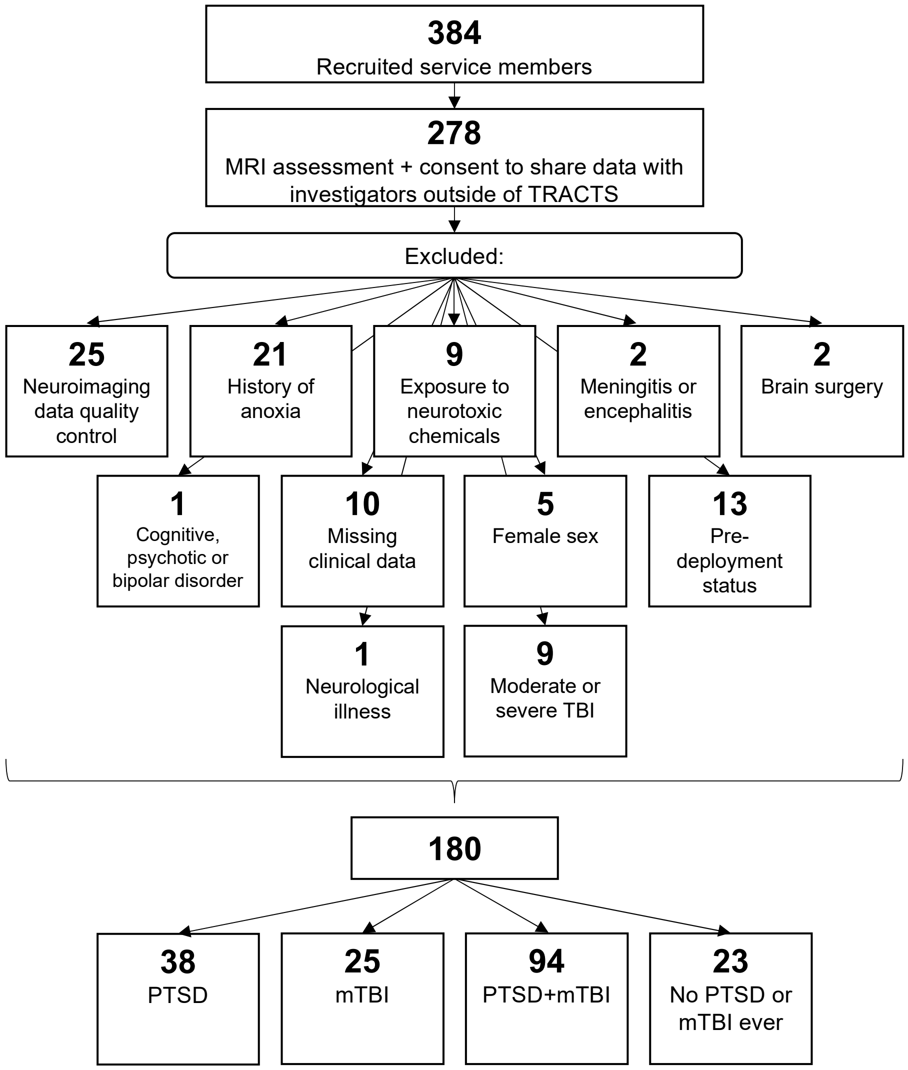

Supplementary Figure S1. Excluded participants and final sample sizes

Note. MRI, Magnetic resonance imaging; TRACTS, Translational Research Center for TBI and Stress Disorders; PTSD, Post-traumatic stress disorder; mTBI, Mild traumatic brain injury. This figure shows the number of excluded participants and the number of the total sample (*N* = 180), and sub-samples (PTSD, *n* = 38; mTBI, *n* = 25; comorbid PTSD+mTBI, *n* = 94; No history of PTSD or mTBI, *n* = 23).

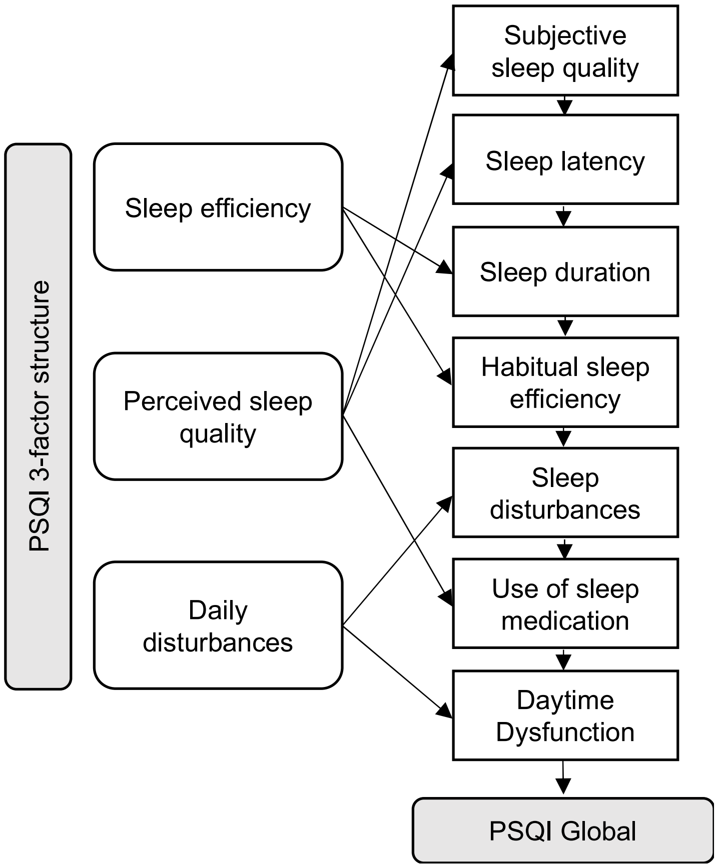

Supplementary Figure S2. Assessment of sleep quality

Note. PSQI, Pittsburgh Sleep Quality Index; PSQI 3-factor structure, The PSQI subscales sleep efficiency, perceived sleep quality & daily disturbances refer to the Pittsburgh Sleep Quality Index 3-Factor Structure.

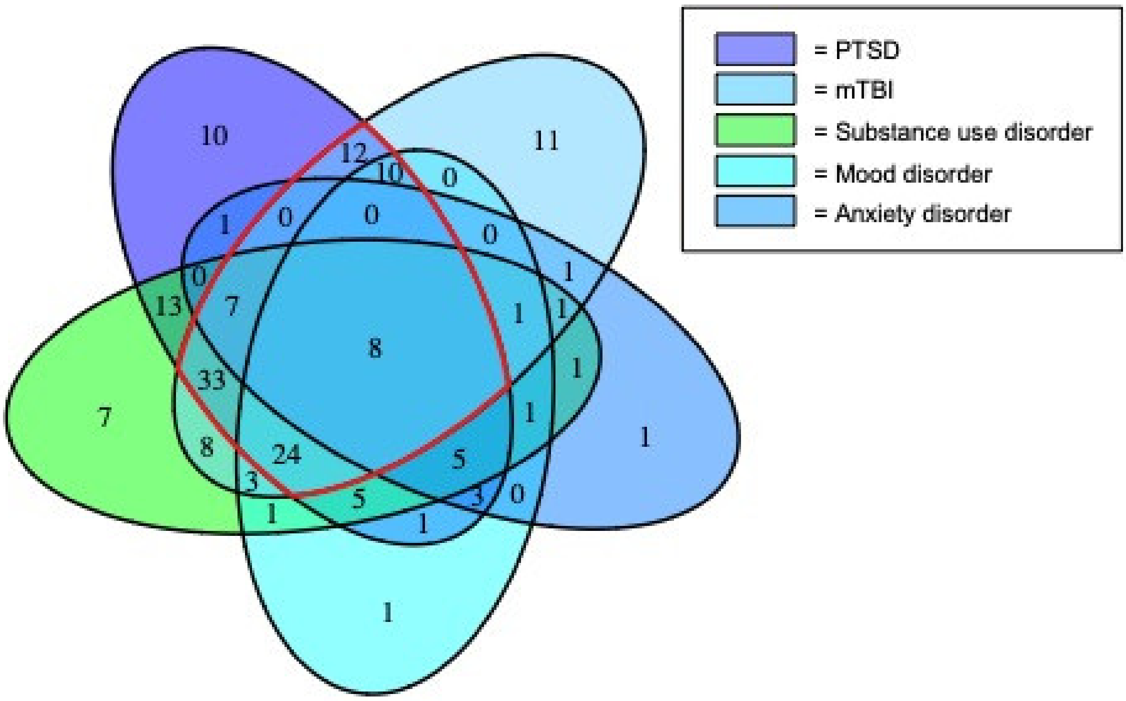

Supplementary Figure S3. Overlap of diagnoses

Note. PTSD, Post-traumatic stress disorder; mTBI, Mild traumatic brain injury. The red-lined area pictures the comorbid PTSD+mTBI sample (*n* = 94) with overlapping substance use, mood and anxiety disorder.

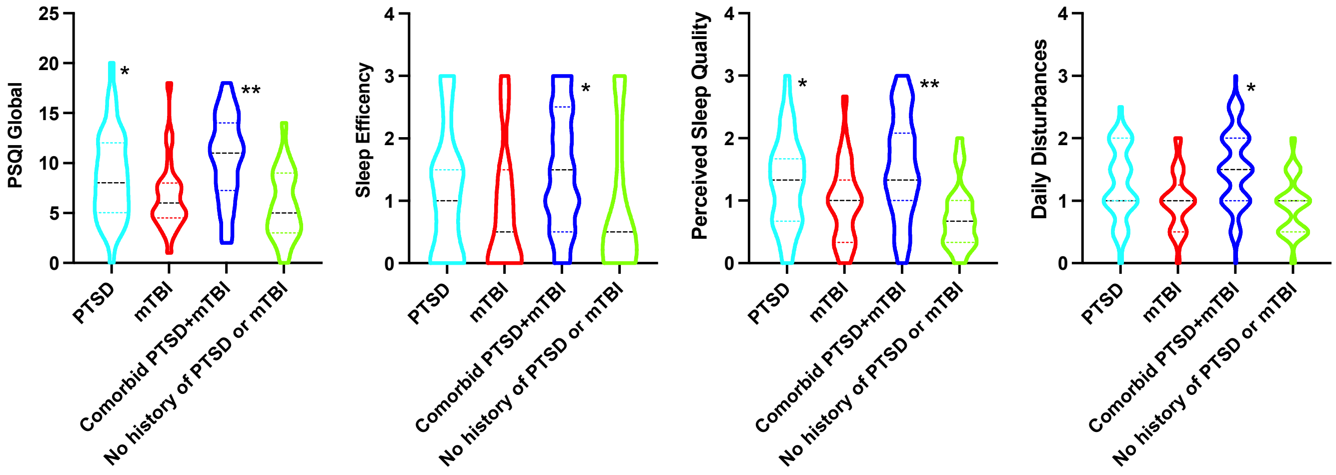

Supplementary Figure S4. Sleep quality group comparisons

Note. PTSD, Post-traumatic stress disorder; mTBI, Mild traumatic brain injury; PSQI, Pittsburgh Sleep Quality Index; The PSQI subscales sleep efficiency, perceived sleep quality & daily disturbances refer to the Pittsburgh Sleep Quality Index 3-Factor structure.

This figure illustrates the significant difference in sleep quality between participants (PTSD vs. mTBI vs. comorbid PTSD+mTBI vs. no history of PTSD or mTBI). Lower scores on the PSQI subscales represent better sleep quality.

PSQI Global: * Significantly higher than no history of PTSD or mTBI. ** Significantly higher than mTBI and no history of PTSD or mTBI.

PSQI Sleep efficiency: * Significantly higher than mTBI and no history of PTSD or mTBI.

PSQI Perceived sleep quality: * Significantly higher than no history of PTSD or mTBI. ** Significantly higher than mTBI and no history of PTSD or mTBI

PSQI Daily disturbances: * Significantly higher than mTBI and no history of PTSD or mTBI.

All analyses are corrected for multiple comparisons as outlined in Figure 1.
